# Improving the health-related quality of life of adult Nigerians living with cancer and their family caregivers: intervention development

**DOI:** 10.1186/s40814-022-01117-w

**Published:** 2022-07-20

**Authors:** Israel Gabriel, Debra Creedy, Amanda McGuire, Elisabeth Coyne

**Affiliations:** 1 School of Nursing and Midwifery, Griffith University, Logan Campus, Meadowbrook, Queensland 4131 Australia; 2 Transforming Maternity Care Collaborative, School of Nursing and Midwifery, Griffith University, Brisbane, Queensland Australia; 3 School of Nursing and Midwifery, Griffith University, Gold Coast Campus, Southport, Queensland Australia

**Keywords:** Psychosocial, Intervention development, Behavioural change, Adults with cancer, Family caregiver, Low-income countries

## Abstract

**Background:**

Evidence for the effectiveness of interventions aimed at improving the health-related quality of life of people living with cancer and/or family members is compelling. However, most interventional research has been conducted in high-income countries, and no intervention had been tested in low-income countries such as Nigeria. It is critical to design a culturally theory-based intervention in a resource-poor setting to address the needs and support coping strategies of cancer patients and their family caregivers.

**Methods:**

Theory, evidence, and practical issues were considered. The Medical Research Council framework for developing and evaluating complex interventions and Behaviour Change Wheel provided the framework for intervention design. Findings generated by a needs assessment of adult Nigerians with cancer and their family caregivers and relevant theories (the Spirituality and the Supportive Care Framework for Cancer) informed content development.

**Results:**

A theory-based, culturally tailored socio-spiritual intervention was developed to address the specific needs of adult Nigerians with cancer and their family caregivers. A 4-week intervention included strategies designed to improve social and spiritual support, information and health literacy, and health-related quality of life.

**Conclusions:**

A systemic approach was used to conceptualise an evidence-based and theory-informed intervention tailored to address previously identified shortfalls in support available to adults living with cancer and their family caregivers, in Nigeria. If implemented and effective, such an intervention has the potential to improve the health-related quality of life of people living with cancer and their families in Nigeria.

## Background

Cancer is a critical public health concern in low- and middle-income countries (LMICs) due to rising obesity rates, increasingly sedentary lifestyles, dietary influences, excessive tobacco and alcohol use, and recurrent carcinogenic infections such as *Helicobacter pylori*, hepatitis B virus, and human papillomavirus [1]. The global cancer burden, morbidity, and mortality are disproportionately higher in LMICs than in high-income countries (HICs), accounting for approximately 70 to 81% of cancer deaths [2, 3].

In Nigeria, an estimated 72,000 cancer deaths occur each year, with 102,000 new cases diagnosed among its 200 million people [4]. The rising burden of cancer in Nigeria places additional strain on already overtaxed healthcare systems and limited economic infrastructure [4]. Cancer diagnosis has been associated with significant psychological distress, as most patients are frequently unprepared to deal with the initial emotional stress caused by the diagnosis [5]. As treatment advances, the patient is confronted with a variety of emotional, spiritual, social, psychological, and physical challenges related to the disease itself, its perceptions, treatment, and caregiving [6, 7]. The prevailing view in Nigeria is that cancer is a death sentence, and many patients believe they have only a slim chance of life [8].

Psychosocial support programmes have predominantly been conducted in HICs [9, 10], with few published studies on the subject from Africa. For example, our systematic review investigating the characteristics and effectiveness of psychosocial interventions on quality of life of adult people with cancer and their family caregivers identified no studies conducted in Africa [11]. Existing interventions may not be applicable to Nigeria for a variety of reasons, including a lack of consideration to spirituality and culture, which are important to people in Nigeria. Little is known about applying interventions in Nigeria where the cancer/caregiving burden is high and demand for resources is great.

In addressing these gaps, it is crucial to design an intervention that integrates information, social, spiritual, and cultural beliefs. Therefore, this study aimed to design an evidence-based intervention to address the needs and improve the health-related quality of life (HRQoL) of adults living with cancer and their families in resource-poor settings such as Nigeria.

## Theoretical framework underpinning the intervention design

This research has been informed by the “gold standard” Medical Research Council (MRC) framework for developing and evaluating complex interventions [12], the most widely used framework in health science [13–16]. The framework recommends beginning with a review of available evidence, followed by a theory phase, and finally a modelling phase [17]. Craig, Dieppe [18] recommended that researchers do a systematic review of the available evidence on the subject, even if a recent optimistic quality review had been conducted. The theory phase entails analyses using theoretical frameworks to develop and model the intervention. The modelling stage involves hypothesising on what should be targeted (determinants of behaviour) and how this can be achieved (via behaviour change techniques) [19].

**Fig. 1:**
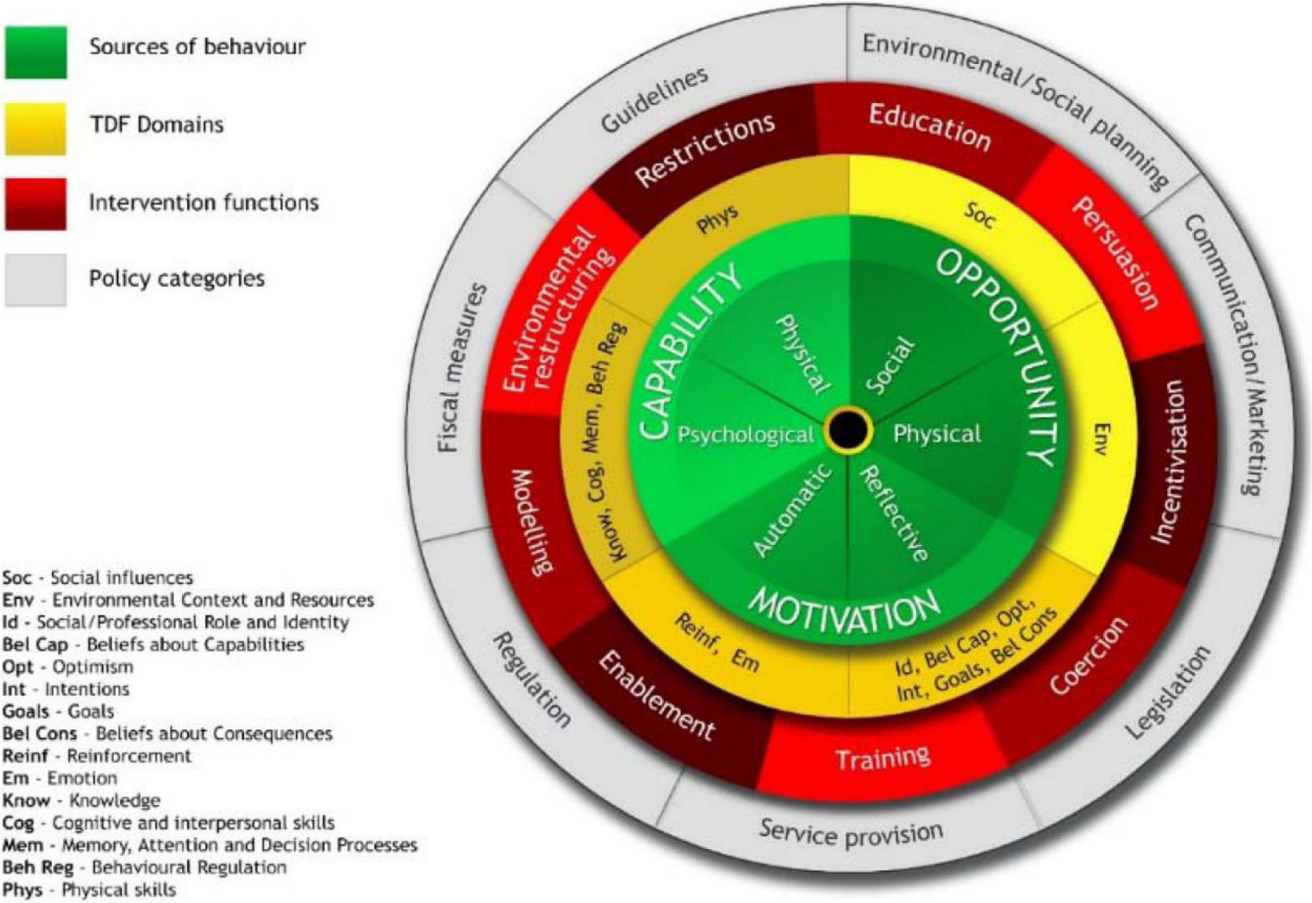
The Behaviour Change Wheel framework

We employed the Behaviour Change Wheel (BCW) [20, 21], which facilitates application of behavioural change theory and has been shown to be extremely effective in developing interventions in order to understand and define target behaviours, identify behaviour change techniques, and specify the intervention strategy and its implementation. The BCW is a systematic guide based on three components: capability, opportunity, and motivation behaviour (COM-B) built around, all of which are necessary to generate or change a behaviour [20] (see Fig. 1). According to COM-B, for any behaviour of an individual, or group to occur, there must be the following: [1] capacity to perform the behaviour, which can be physical or psychological [2]; the opportunity for the behaviour to occur, which can be physical, for example environmental factors that allow or promote behaviour, such as time, resources, stimuli, or social (e.g. opportunity afforded by interpersonal influences, social cues, and cultural norms); and [3] motivation to perform the behaviour at the relevant time, which can be reflective (e.g. requiring self-aware planning and evaluation) or automatic (e.g. processes involving wants, needs, desires, impulses, and reflex responses) [20–22].

The BCW has nine intervention functions and seven policy-level strategies used to link influences on behaviour, identified by the COM-B, to potential intervention functions and policy categories.

## Methods

**Fig. 2:**
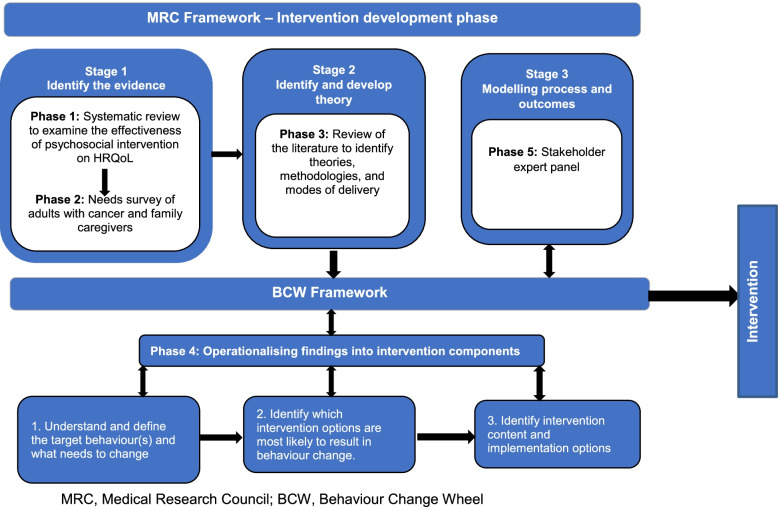
Intervention design process. MRC, Medical Research Council; BCW, Behaviour Change Wheel

Development of the theory-based, culturally tailored intervention for adult Nigerians living with cancer and their family caregivers consisted of five phases, which are broadly categorised into 3 stages (see Fig. 2).

The five phases are as follows:

1. A review of literature to establish the characteristics of psychosocial interventions for people living with cancer and their family caregivers. This is detailed in stage 1 of the MRC framework.
2. Assessment of needs and HRQoL of adult Nigerians living with cancer and their family caregivers. It is described in stage 1 of the MRC Framework.
3. Theoretical framework selection and application are covered in stage 2 of the MRC framework.
4. Operationalising findings into intervention components. This is detailed in the MRC framework stage 2.
5. Process and outcome modelling are outlined in stage 3 of the MRC framework.

The methods and results of phases 1 and 2 have been published elsewhere [11, 23]; hence, they are only briefly summarised below.

### MRC framework stage 1 — identifying the evidence base

To change behaviour, one must first understand why certain behaviours occur and what must change for the desired behaviour to occur [24]. Thus, we examined the evidence from previous studies to identify important behaviours to inform the primary outcome of behaviour change [25, 26].

#### Review of literature

The systematic review of the literature aimed to (i) examine the characteristics of the psychosocial interventions and (ii) examine the effectiveness of the psychosocial interventions on HRQoL of people with cancer and their family caregivers (PROSPERO CRD42020144563).

#### Needs assessment

Adult Nigerians with cancer and their family caregivers who were attending an oncology clinic for cancer management were invited to take part in the needs assessment. The survey looked at physical, psychological, family/social, spiritual, and healthcare staff needs, as well as information, practical support, cancer health literacy, and HRQoL.

### MRC framework stage 2 — identify/develop theory

#### Developing a theoretical underpinning for the intervention

By incorporating theory into the design of behaviour change programmes [27], we can better understand the causal pathways by which an intervention modifies health behaviour. This stage involves identifying relevant theories that provide insight into the mechanisms through which an intervention is likely to influence individual behaviour. Theoretical frameworks, including the Spirituality Framework [28] and the Supportive Care Framework for Cancer [29], were reviewed, while the MRC framework and BCW were selected to guide the intervention development process [12, 20, 30].

**Fig. 3:**
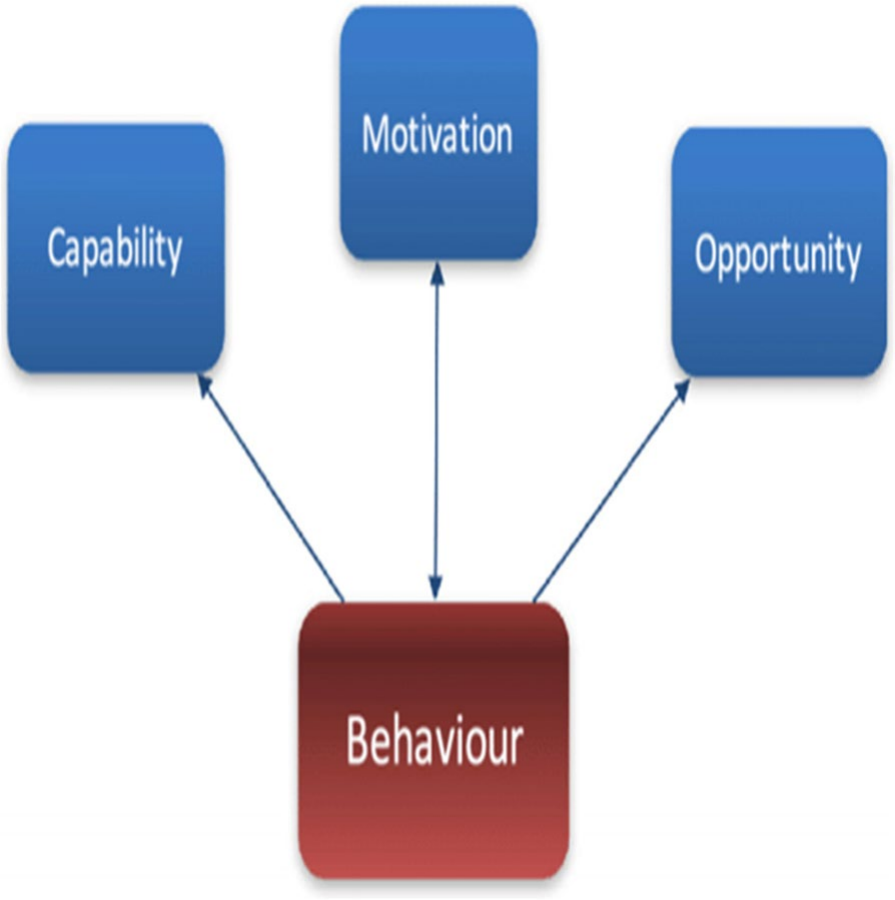
Model of Behaviour Change (COM-B). The relationship between capacity, opportunity, motivation, and behaviour

Firstly, the BCW process involved defining the problem in behavioural terms and selecting the target population and behaviour. A behavioural diagnosis was conducted, which included findings from the needs assessment and the theoretical frameworks with COM-B model (see Fig. 3) constructs to determine what strategies should be implemented to support target behaviour performance. Factors identified in the COM-B behavioural diagnosis were further subdivided into 14 theoretical domains according to the Theoretical Domains Framework (TDF) which is recommended as part of the BCW method [20, 30, 31].

Secondly, elements of the behavioural diagnosis were linked with intervention functions (e.g. education, persuasion, training) likely to influence the target behaviours. For instance, education may be used by interventions to teach cancer parents about coping with cancer trajectory, and training equips them with positive coping skills. APEASE criteria (affordability, practicability, effectiveness/cost-effectiveness, acceptability, safety, and equity) [20] were applied to each intervention function to determine its appropriateness for the needs context.

Finally, the BCT taxonomy [32] was used to identify possible behaviour change techniques that best served the intervention functions. The final stage of the intervention design process was to determine the most effective delivery mode. The selected behaviour change techniques and mode of delivery were translated into intervention components. We conducted a mapping exercise to outline the links between intervention components, intervention functions, Theoretical Domains Framework constructs, and behaviour change techniques. The key findings from each of these BCW steps were synthesised.

### MRC framework stage 3 — modelling the process

The first author then organised stakeholder engagement meetings in Nigeria with three oncology nursing experts and five adults with cancer/family caregiver dyads to consider the need assessment findings and intervention material.

#### Operationalising findings into intervention components

The intervention content and format were developed based on the information gained by the preceding stages (i.e. as reported under the MRC stages 1 and 2 headings).

## Results

The results from each stage of the intervention development process are outlined below.

### MRC framework stage 1 — identifying the evidence base

According to the findings of the systematic literature review, psychosocial interventions are effective in improving the HRQoL of people living with cancer and their family caregivers [11]. The 12 studies included primarily focused on psychological, physical, and social domains of HRQoL, with spiritual well-being receiving little attention despite its effect on HRQoL. While there is evidence that psychosocial interventions have a significant positive effect on HRQoL, no interventional research aimed at improving HQoL has been conducted in Nigeria among people with cancer and their family caregivers.

We conducted a needs assessment using a cross-sectional descriptive approach with 120 adult Nigerians with cancer and their family caregiver dyads [23]. The findings of the needs survey and systematic literature review produced four evidence statements, which are as follows:

- *Evidence statement 1*: The negative impact of cancer diagnosis and caregiving on adult Nigerians with cancer and family caregivers’ HRQoL domains (spiritual, social, physical, and psychological)
- *Evidence statement 2*: Cancer information/health literacy is a central factor in understanding cancer progression and treatment options.
- *Evidence statement 3*: Spiritual and social support may improve HRQoL of adults with cancer and their family caregivers.
- *Evidence statement 4*: Family caregivers face considerable challenges in relation to the caregiving role added to their primary responsibilities.

### MRC framework stage 2 — application of theoretical framework to inform intervention development

Following completion of stage 1 of the BCW process, a comprehensive behavioural diagnosis was conducted using the COM-B model. The exercise identified all six sources of behaviour change on the BCW. Table 1 presents the findings of the COM-B model-guided behavioural diagnosis. This provides evidence statements in relation to physical capacity, psychological capability, physical opportunity, social opportunity, reflective motivation, and automatic motivation that should be considered when developing the intervention to address the needs and improve the HRQoL of adult Nigerians living with cancer and their family caregivers.

**Table 1:** Behavioural diagnosis using the COM-B model

COM-B component	Behavioural diagnosis
Physical capability	╸Physical strength is needed to engage in religious and community activities ╸A need for physical strength to participate in training and to meet people with similar conditions to share experiences
Psychological capability	-Need for socio-spiritual awareness that will dispel myths about cancer and caregiving ╸Need knowledge of how best to promote healthy behaviour in adults with cancer and family caregivers
Physical opportunity	╸Adults with cancer and family caregivers need the resources that will meet their needs and thereby improve their quality of life - Adults with cancer and family caregivers have limited time for engagement ╸Adults with cancer and family caregivers need access to safe environment and facilities ╸Adults with cancer and their families find ways to overcome any financial and time constraints that prevent them from receiving care
Social opportunity	╸Adults with cancer and family caregivers need social support from friends and family ╸During the disease/caregiving journey, adults with cancer and family caregivers need opportunities to engage with friends, relatives, and significant others ╸Adequate support from healthcare providers ╸Family members encouragement is crucial ╸Continued maintenance of logistic support from government and society should be encouraged ╸Social group participation should be encouraged
Reflective motivation	╸Adults with cancer and family caregivers need to develop a habit getting involve in the community events that will promote their quality of life ╸Adults with cancer and family caregivers need to develop plans for daily/weekly activities and develop a habit of participation
Automatic motivation	╸Adults with cancer and family caregivers need to feel that they want to participate in socio-spiritual activities, and that there may be a sense of pleasure or satisfaction from participation ╸Family members may feel a sense of duty to set a good example for their cancer-affected loved ones and caregivers, and this may be motivated by a desire to do the best for loved ones

*COM-B* Capability, opportunity, and motivation behaviour

According to Michie, Van Stralen [33], capability, opportunity, and motivation interact to generate behaviour (see Fig. 3). The ability of a person to perform an activity is described as their psychological and physical capacity [33]. It entails possessing the required skills and knowledge [33]. The cognitive states that energise and guide behaviour are collectively referred to as motivation. It includes habitual processes, emotional responding, and analytical decision-making [33]. Opportunity is defined as all the factors that lie outside the individual that make the behaviour possible or prompt it [33]. In consideration of the four evidence statements and the numerous behaviour diagnoses regarding capability, opportunity, and motivation (see Table 1), two target behaviours capable of influencing other behaviours were identified: [1] involvement in socio-spiritual behaviours to increase HRQoL across the study population and [2] appropriate knowledge of the disease process and caregiving among adults with cancer and family caregivers.

These are supported by the Spirituality Framework and the Supportive Care Framework for Cancer, both of which highlight the importance of spiritual, social, information, psychological, or physical support in improving the HRQoL of cancer patients and their family caregivers [28, 34].

The nine intervention functions to prompt behaviour change were identified from the BCW. The intervention functions were graded according to the APEASE criteria. Two intervention functions (coercion and restriction) were eliminated as they did not meet APEASE criteria. The following seven intervention functions were selected: education, persuasion, incentivisation, environmental restructuring, training, modelling, and enablement. Eight out of the fourteen TDF domains were identified during the analysis as influencing the seven identified behaviours. These eight domains were as follows: skills, knowledge, behavioural regulation, social influences, environmental context and resources, professional/social role and identity, emotion, and reinforcement. Each was a target of the intervention (see Table 2).

**Table 2:** Analysis of target behaviours impacting positive behaviour using COM-B and TDF

COM-B	TDF Domain	Relevance of domain	Intervention function(s)	Behaviour change technique(s)	Description of the intervention's behaviour change technique
Physical capability	Physical skills Emotion	Individuals do not have the skills essential to cope with a cancer diagnosis and to provide cancer care	Training	Instructions on how to perform specific behaviours such as breathing exercise	Advise on how to adjust their existing behaviour in accordance with the guidelines
				Demonstration of the behaviour	Individuals will be introduced to resources, such as graphical instructions, and encouraged to demonstrate caregiving abilities
				Behavioural practice/rehearsal	Prompt participants to practise certain tasks throughout the intervention
		Participants multitasking skills	Modelling	Graded tasks.	Adults with cancer + family caregivers encouraged to achieve simple tasks before graduating to more complex duties. Highlight importance of self-care
				Self-monitoring of behaviour	Individuals urged to keep weekly reflection notebooks to track their progress in developing their skills
Physiological capability	Knowledge	Individuals do not understand or have wrong knowledge of the guidelines Intergenerational transmission of insufficient and/or inaccurate knowledge	Education	Instruction on how to perform behaviour	Individuals will be given extensive verbal and written information about the behavioural guidelines, as well as the opportunity to ask any questions they may have — to avoid misinformation from being spread. Performed during the intervention session Individuals will be given comprehensive information on cancer symptoms, management, and complications Baseline data will be analysed prior to intervention to identify any pre-existing beliefs/behaviours indicative of misinformation. This will be brought up in a proactive manner for discussion with participants
	Behavioural regulation	Individuals find it challenging to self-regulate their behaviour because they perceive new guidelines to be restrictive and difficult to implement into their daily lives	Enablement	Self-monitoring of behaviour	Individuals are requested to keep weekly reflection records in which they track their progress towards achieving the goals specified for desired behaviours that will be discussed during the intervention
				Goal setting (behavioural)	The goal is to successfully address the social, spiritual, and information needs and improve the quality of life of adults living with cancer and their family caregivers and strengthen their knowledge and skills to mobilise social networks Individuals are prompted to develop their own goals that they believe are attainable — goals that take into account their preferences and willingness to change. The goals will be SMART
				Action planning	Individuals will be urged to make detailed plans outlining how they intend to accomplish each goal
				Prompts and cues	Individuals are encouraged to leave the intervention booklet in a frequented location to encourage them to read it and participate in the agreed-upon actions
				Information about emotional consequences	Facilitators should communicate with individuals the potential for improved mood if they choose to engage in desirable behaviours
Social opportunity	Social influences and emotion	It is easier to adopt a new behaviour when surrounded by family members who are also doing so	Enablement	Social/spiritual support (practical)	Individuals are advised to seek assistance from family members (including those who are not participating in the intervention) and friends if they are having difficulty engaging in the desired behaviours Encourage participants to read spiritual writings, such as the Bible, Koran, or other faith-based texts. Seek the assistance of others; for example, you could initiate an ongoing dialogue with your clergy or counsellor, or you could join a community for meditation, prayer, and support, as well as listening to classical or spiritual music
				Problem-solving	During the “barriers and solutions” section of the intervention, facilitators will encourage participants to consider scenarios in which they believe they will struggle to engage in desirable behaviours (social situations) and devise strategies to overcome these obstacles. These will be discussed further during follow-up sessions
			Environmental restructuring	Restructuring of physical and social environment	Individuals are encouraged to socialise with friends and family in settings that enhance the participation of desired behaviours, such as a church, mosque, or park
Physical opportunity	Environmental context and resources	Life circumstances that such as illness or a new job can diminish their available capacity/resources/time to follow the guidelines	Training	Instruction on how to perform the behaviour	The instructions given by facilitators to individuals will be tailored to their specific care needs and other conflicting occurrences in their lives that require their attention. Individuals will receive instructions on how to achieve the desired behaviours in their existing situation
		A scarcity of resources such as finances and infrastructure in the environment	Incentivisation Enablement	Social support (emotional)	Facilitators will provide emotional support to participants during intervention sessions by discussing what else is going on in their lives and how this is affecting their ability to adhere to guidelines. Individuals are also encouraged to seek emotional support from friends and relatives
Reflective motivation	Professional/social role and identity		Persuasion	Framing/reframing	Individuals are communicated with cancer-specific guidelines, as opposed to general health guidelines that are offered to everyone. The benefits of following cancer management and caregiving suggestions, in addition to the benefits to general health, will be emphasised
				Verbal persuasion about capabilities	Tell the person that they can successfully perform the wanted behaviour, arguing against self-doubts, and asserting that they can and will succeed
				Feedback on outcomes of behaviour	Individuals will be evaluated at the start and end of the intervention on their ability to adhere to the guidelines and maintain their behaviour after the intervention
			Modelling	Credible sources	The intervention will be guided by facilitators who will detail the training they went through to get that title — to help individuals, recognise the validity of their advice. Furthermore, all individuals will be informed that their physician/nurse is aware of and supportive of them receiving the intervention as part of their clinical care
				Review goal (behavioural)	Each session will begin with a recap of the previous session’s goals. Participants will determine whether to keep the current goal, modify it, or create a new one. These decisions will be made based on an individual’s level of achievement and willingness to change
Automatic motivation	Reinforcement	Participants are incentivised (subsidy from the government) where appropriate to ensure that they follow instructions and achieve the best possible health outcomes	Persuasion	Comparative imaging of future outcomes	Facilitators should urge people to think about the potential health consequences of following recommendations vs not following them, with a focus on the potential health effects
			Enablement	Social support (emotional)	Facilitators should encourage participants to offer support to one another and to engage in the behaviours throughout the session

*SMART*, specific, measurable, acceptable, realistic, and time based

The salient COM-B and TDF constructs were mapped to seven intervention functions and 19 behaviour change techniques included in the intervention (see Table 2). For example, during a supervised breathing exercise, group facilitators could demonstrate breathing techniques and then provide feedback to participants regarding their technique. As such, the supervised breathing exercise was mapped to the behaviour change techniques 4.1 “Instruction on how to perform the behaviour” and 2.2 “Feedback on the behaviour” (as shown in Table 2).

### MRC framework stage 3 — stakeholder expert panel findings

The expert panel proposed a number of recommendations to optimise intervention implementation and impact, including the importance of cancer health literacy, as most people living with cancer and their family caregivers may hold one or more cancer myths that impact cancer outcomes. The importance of cultural and religious beliefs was also highlighted. They also argued that content should be written in simple language, free of medical terms that may be unfamiliar to participants. Adults with cancer and their family caregivers advocated for a small, easy-to-read booklet they could take home to reinforce key information and helpful behaviour change. Protocols were also developed to clearly describe how to address issues raised by participants that were outside the scope of the intervention. This included identifying outside support services to which participants could be directed and/or referred. To address this and ensure intervention fidelity, facilitators involved in intervention delivery received online training and ongoing supervision from the first author.

#### Mode of delivery

The BCW recommends that all alternative forms of intervention delivery are explored before selecting the most appropriate approach for the target behaviour, population group, and setting [20]. A four-module interactive, face-to-face training programme was developed. The selection was based on the evaluation against the APEASE criteria. The training was planned to be conducted in four, 120-min sessions. Intervention materials were developed, alongside training manuals for participants and facilitator manuals. Table 3 summarises the planned content and goals, as well as the behaviour change techniques incorporated into each session. Additional file 1 describes the full content of the training for facilitators.

**Table 3:** Breakdown of intervention content with BCTs

Sessions	Goal(s)	Group processes	Incorporated BCTs
Session 1	To appreciate the experience and common concerns of people affected by cancer across the cancer journey To describe the strategies employed in the local setting to promote approaches to care	Help participants share fears and concerns about cancer and its treatment Educate about illness and treatments and caregiving according to identified knowledge gaps and misconceptions based on participant feedback Encourage optimistic thinking Communicate and demonstrate a range of possible therapies such as relaxation, massage Encourage healthy coping and lifestyle behaviours	Instruction on how to perform the behaviour, credible sources, information about emotional consequences
Session 2	To encourage the expression of feelings and ideas To assess verbal and nonverbal client communication needs To respect the client’s personal values and beliefs	Ask participants to write down their concerns and fears about cancer prognosis Discuss results among the group Promote open communication Encourage mutual support and teamwork Promote approaches to helping dyad work within the limits of their new limitations	Instruction on how to perform behaviour, action planning, demonstration of the behaviour, behavioural practice/rehearsal, framing/reframing, social support (physical and emotional)
Session 3	To provide relief from suffering, manage symptoms, help reconcile relationships, and assist in the transition between this life	Help dyad to stay hopeful in the face of death through honest conversations with family and close friends Social connections; link between the past and present Help dyad deal with overwhelming stress by talking to someone they trust, exercising, and making time for activities they enjoy	Social (physical, emotional) support, problem solving, restructuring of physical and social environment, verbal persuasion about capabilities
Session 4	To recognise family’s positive attributes Develop family coping during challenging times	Identify family strengths Use reflective writing to deepen insights and reflect on life changes and what is essential in life Discuss the use of spiritual coping with health challenges Consider what provides a sense of inner peace for the individual Explore the views of each other and come to a new shared understanding	Social (physical, emotional) support, behavioural practice/rehearsal, graded tasks, verbal persuasion about capabilities, feedback on outcomes of behaviour
Wrap-up and instructions	Address any unresolved issues/questions Provide encouragement and motivation Review the weekly reflection notebook and consider progress		Social support (emotional), review goal (behavioural), verbal persuasion about capabilities

## Discussion

The systematic approach to the design process led to the development of a novel intervention that is patient/family caregiver-centred, evidenced-based, and theoretically informed.

The behaviour change techniques identified in this study were derived from quantitative data and theoretical sources. The COM-B/TDF model was used to quantitatively analyse sources of behaviour, which was then linked to subsequent intervention functions within the BCW, and, finally, identify the appropriate behaviour change techniques to use when developing a tailored intervention to meet the needs of adult Nigerians living with cancer and their family caregivers.

This study identified physical and psychological capability, social and physical opportunity, and reflective and automatic motivation as key targets for a behaviour change intervention for addressing the needs of the study population. In addition, physical skills, knowledge, behavioural regulation, social/spiritual influences, environmental context and resources, social role and identity, emotion, and reinforcement were important TDF domains that need to be targeted in the intervention. Consequently, seven intervention functions including education, persuasion, incentivisation, training, environmental restructuring, modelling, and enablement were identified as relevant for the intervention (as shown in Table 2). These findings were in accordance with the MedEx IMPACT intervention study by Cantwell, Walsh [35] in which the BCW was used to design a physical activity behaviour change intervention for individuals living with cancer. Cantwell, Walsh [35] selected five intervention functions for their study, whereas the current study identified a broader range of intervention functions because it used the APEASE criteria to determine the most relevant intervention function for the target population.

This study utilised 19 behaviour change techniques identified from this process. Targeting cognitive memory by providing prompts and cues, rewarding successful completion of the target behaviour, and providing information about community misconceptions and the consequences of such misconceptions about cancer are just a few of the strategies that can be used to implement behaviour change techniques.

While the BCW and MRC framework offered structured direction for intervention development, there are significant gaps within this guidance. Firstly, both approaches highlight the importance of theory in developing interventions, but neither offer explicit guidance on how to select and apply appropriate theories especially when selecting behaviour change techniques [36]. To tackle this issue, we had to review potentially relevant theories, which were selected to underpin this intervention. Linking theory and intervention content is challenging and may explain why many interventions are developed without a relevant theory [37]. Further research is required to map constructs derived from theories onto effective intervention content [27]. Researchers should consider both theoretically derived behaviour change techniques and those generated by the target population.

The development of the intervention advances implementation science and intervention design research by providing a detailed account of the intervention development process and clearly outlining how theory has informed and been embedded within an intervention for people with cancer and their family caregivers. The training package is reported to the point where it can be delivered in a feasibility study. Testing the integration of intervention components is a critical part of intervention development, and it is expected that further refinements may be made before piloting and full evaluation of the intervention in a randomised controlled trial.

A major strength of the study is the systematic approach used to develop the socio-spiritual intervention, which allows for a rigorous evaluation of the intervention. Employing an integrative theoretical approach guarantees that all components of the intervention will optimise future benefits for participants. Although the BCW is viewed as strongly optimistic and helpful in guiding intervention development, it is not without limitations. The process was time-consuming, from problem identification to intervention design.

## Conclusions

This study demonstrates the evidence and theory used to inform the development of a psychosocial intervention. The MRC framework and BCW processes provided a systematic, pragmatic, methodical, and complementary approach to designing a theory-based intervention to meet the needs of adults living with cancer and their family caregivers in Nigeria. The synthesis of findings from this formative research has resulted in the development of a novel intervention that aims to address the needs of this population in Nigeria. The next phase in this research is to evaluate the feasibility and preliminary effectiveness of the intervention among adult Nigerians living with cancer and their family caregivers.

## Abbreviations

COM-B: Capability, opportunity, and motivation-behaviour
LMICs: Low- and middle-income countries
HRQoL: Health-related quality of life
HICs: High-income countries
MRC: Medical Research Council
BCW: Behaviour Change Wheel
RCT: Randomised controlled trial
APEASE: Affordable, practical, effective/cost-effective, acceptable, safe, and equitable
BCTs: Behaviour change techniques
TDF: Theoretical Domains Framework
